# The relationship between agility and vertical jump performance in male handball players: A cross-sectional analysis on the role of developmental characteristics

**DOI:** 10.1371/journal.pone.0333203

**Published:** 2025-09-17

**Authors:** Rıdvan Ergin, Betül Karcı

**Affiliations:** 1 Faculty of Sports Sciences, Department of Physical Education and Sports, Recep Tayyip Erdogan University, Rize, Türkiye; 2 Recep Tayyip Erdogan University, Institute of Graduate Studies, Physical Education and Sports Master’s Program with Thesis (Master’s Degree Advisory Student), Rize, Türkiye; Erzurum Technical University: Erzurum Teknik Universitesi, TÜRKIYE

## Abstract

**Aim:**

This research examines the relationship between agility and vertical jump by controlling for developmental characteristics (age, height, and body weight), which are important for sports performance yet underexplored in literature.

**Materials and methods:**

In this quantitative research, the study group was determined by power analysis and participants were selected using purposive sampling method. The study group consists of 15 male handball players (age 24.1 ± 5 years) who volunteered to participate. Agility performance was determined by the *“Illinois Test”*, while jumping performance was assessed by the *“Vertical Jump Test”*. The *Pearson* test was used in the relationship analysis as a result of the normality assumption. Additionally, a partial correlation analysis was conducted to control for the effect of developmental characteristics (α = 0.05).

**Results:**

A positive significant relationship was found between age and years of sports (r = .759; p < 0.01) and vertical jump (r = .657; p < 0.01) in male handball players, as well as a positive significant relationship between body weight and agility (r = .621; p < 0.05). Additionally, while controlling for age (pr = −.557; p = 0.039) and body weight (pr = −.558; p = 0.038), a negative significant relationship was found between agility and vertical jump.

**Conclusion and suggestions:**

As a result, based on the studies examined, it has been observed that male handball players have a high level of agility and vertical jump performance. At the same time, it has been noted that a decrease in age increases agility performance, while an increase in age enhances jump height. The statistical relationship between agility and vertical jump has only been determined when developmental characteristics are controlled. Therefore, it is suggested that to better clarify the relationship between variables in sports performance research, studies should be conducted with developmental characteristics controlled. Additionally, trainers should train agility and vertical jump, testing athletes regularly at set intervals throughout the season for performance tracking.

## Introduction

In handball, both offense and defense generally occur on the same line. At the same time, there is a change of position and direction while advancing from the defensive area to the attacking line. Therefore, handball players may need to make sudden movements and reposition themselves during these movements. This information highlights the concept of position and direction change, that is, agility [1]. From another perspective, looking at the nature of the game in the sport of handball, it is evident that high jumping during the game provides players an advantage not only in blocking shots in defense but also in delivering the ball over the opponent to the goal (shooting) [2]. It is known that pivot, wing, and playmaker position handball players use the jump shot technique [3]. Due to the nature of the handball game and the jumping shooting technique, this research focuses on agility and vertical jump performance.

In the century we are living in, developed or developing countries are trying to assert their superiority over each other in sports competitions, and therefore athletes are pushing their performance limits. In fact, this situation can create a perception of modern athletes as warriors due to their pushing the limits of high performance. This perception is also supported by the frequently used metaphor of *“sport=war”* in sports media [4]. Thus, athletes, regardless of the sport they are interested in, want to prove their success with high athletic performance. It is emphasized that agility performance is important for high athletic performance, and agility is defined as the coordinated change of place and direction, kept under control [5]. Therefore, in all sports, including handball, agility performance stands out in athletes during transitions from defense to offense and in movements to the left and right in both defensive and offensive areas.

In the sport of handball, agility performance is accompanied by high-intensity activities such as jumping (during shots and blocks [2]) [6]. These high-intensity activities indicate the effectiveness of the anaerobic system. The anaerobic system emphasizes explosive power performance. For this reason, agility and vertical jump tests can be expressed as indicators of explosive power performance [7]. Thus, performance characteristics that are important for many sports and specifically for handball need to be determined through field tests. Additionally, while creating agility training programs, different indicators of agility performance, such as explosive power (vertical jump, etc.), should be tested. As a result of this test, it should be analyzed whether there is a statistical relationship with agility performance [8]. During these analyses, developmental variables should be identified. By keeping developmental variables under control, the statistical relationship between the performance parameters to be examined becomes clearer [9]. Thus, the performance parameters of handball players can be more clearly defined, and their performance levels can be analyzed and monitored without being affected by developmental or maturity variables. Additionally, performance parameters that are related or affect each other are also identified. Therefore, alongside individual performance tracking, other performance characteristics that are associated with or affect the desired performance trait can be included in training programs, facilitating multi-faceted development of sports performance.

Although agility and vertical jump performance, which are among the fundamental components of explosive strength, are addressed separately in some studies, both characteristics are based on neuromuscular coordination and the anaerobic energy system. In vertical jumping, the force production of the lower extremity is involved, while in agility performance, the athlete’s rapid changes in place and direction are present [10–12]. Therefore, both performance components require high-speed muscle contractions and quick reaction times. Despite the close relationship between the concepts, studies examining the relationship between the two performance characteristics, while controlling for developmental factors (age, height, and body weight), are quite limited. For this reason, clarifying whether the relationship between the two performance components develops together or independently will contribute to the literature.

The main aim of this research is to determine the agility and vertical jump performance of male handball players and to examine the statistical relationship between these performance characteristics. Due to the dynamic nature of the handball game, players may need to continuously change direction during the transition from defense to offense, and at the moment of attack, especially in front of the defense line, they may have to jump in order to surpass the defense and shoot to connect the ball with the goal. Therefore, in order to better and more accurately understand the relationship between these two important performance characteristics, variables such as age, height, and body weight, which are developmental characteristics of handball players, will be controlled during the analysis. Thus, the relationship between agility and vertical jump of handball players will be evaluated independently of individual physical characteristics. In line with this purpose, the following hypotheses have been established for the execution of the research.

**H**_**1**_: There is a statistically significant relationship between agility and vertical jump performance.

**H**_**2**_: Agility and vertical jump performance are significantly related when controlling for age, height, and body weight.

## Materials and methods

### Model of the research

**Fig 1:**
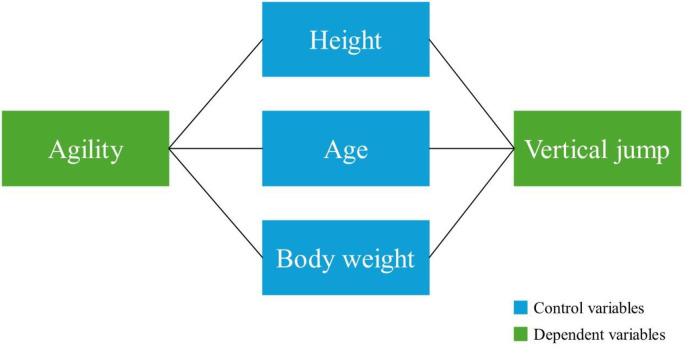
Conceptual model created for research.

In this research, a relational scanning model was used to determine the relationship between variables through a quantitative approach. Agility and vertical jump performance were considered as dependent variables in the research. To clarify the relationship between the dependent variables, developmental characteristics (age, height, and body weight) were included as control variables in the model. This control was achieved through partial correlation (pr) analysis. The conceptual model created for the research is shown in Fig 1.

### Study group

According to the purposive sampling method, inclusion criteria have been established by researchers for determining the study group. These criteria include being a male handball player, being a competitive athlete, participating regularly in training sessions, being healthy, and voluntarily participating in the research. In addition, a study similar to this one [13] was examined for determining the study group. Based on the relevant study data (Table 4), a power analysis (G*Power 3.1, Germany) was conducted, resulting in (Tail(s)=Two, Correlation ρ H1 = 0.8185353, α = 0.05 and Power (1-β)=0.95), which indicated that 12 handball players are sufficient for this research [14]. In the province of Rize, there are approximately 30 handball players who meet the established criteria, and data was collected from 21 handball players who voluntarily participated. Among these handball players, 6 were excluded from the study because they did not meet the inclusion criteria (regular participation in training). Therefore, based on the purposive sampling method and the result of power analysis, the study group of the research, consisting of voluntary participants, is made up of 15 male handball players (age 24.1 ± 5 years) from 1st league men’s handball teams (n = 11; Güneysu Sports Club and n = 4; Rize Municipality Sports Club) who played in the 2024–2025 season.

### Ethics and permissions

The ethical standards of Recep Tayyip Erdoğan University and the Helsinki Declaration (revised in 2013) have been taken into account for the method applied in this research. Additionally, the *“Rize Recep Tayyip Erdoğan University Social and Human Sciences Ethics Committee (Meeting Date: 17.01.2024; Meeting Decision Number: 2024/039)”* has approved the research. Personal information of the participants is not included in the study. An oral notification and written consent (Informed Consent Form) have been obtained to confirm that the participants voluntarily participated in the research. Furthermore, permission has been obtained from the relevant clubs to conduct the research.

### Data collection tools

In the data collection tools, there is a *“Personal Information Form”* created by researchers for the demographic characteristics of male handball players, which includes relevant tests to measure height, body weight, agility, and vertical jump performance. Additionally, data collection and data organization processes from male handball players were carried out between April 8 and April 16, 2025.

### Personal ınformation form

In this form, there are questions and data related to the variables of male handball players; age (year), height [cm (centimeter)], body weight [kg (kilogram)], sports year (year), vertical jump (cm) and agility [sec. (second)].

### Height and body weight

A tape measure with a precision of 0.01 m (meter) was used to measure the height of the study group. When determining height, attention was paid to ensuring that male handball players were on a flat surface, without shoes and in a standing position. Heights were recorded in centimeters. For measuring the body weight of the study group, a digital scale (Xinghang, China) with a precision of 0.01 kg was used. During the measurements, it was ensured that male handball players were without shoes and wore light clothing that would not affect the measurement results [15].

It was stated that before the tests were conducted, the team coaches should be informed so that the male handball players are not tired, they have had their sleep, and they should finish their eating and drinking processes 2 hours before the test. The tests used were explained verbally and the opportunity to practice was provided. After measuring the height and body weight of the male handball players, each participant was given a 10-minute dynamic (light jogging, mobility exercises, and low-intensity jumping exercises) warm-up period before the tests [16]. The handball players first performed a vertical jump test followed by an agility test. A fixed rest period of 5 minutes (3 minutes passive rest; 2 minutes active rest) was provided between tests and repetitions [17]. The measurements were conducted by the same experienced researcher. The researcher provided the necessary commands and motivational phrases (like ‘well done’, ‘faster’) to the handball players before and during the tests. The tests were conducted between 3:00 PM and 6:00 PM in an indoor space with a parquet floor and at an ambient temperature of approximately 22–24 °C [18–19].

### Vertical jump test

The vertical jump height of male handball players has been determined using a vertical jump measurement device (Takei Jump Meter, Japan). This device features a circular black mat laid on the ground, with a cord extending from the center of the mat to a measuring apparatus that will be attached to the player’s waist with a belt. After warming up, the handball player steps onto the mat, and the cord is tightened to zero the measurement value of the device. The handball players then jump upwards with all their strength when they feel ready, with their hands on their hips and their knees bent at 90°. The measurement method was repeated twice, and the best result was used for analysis in centimeters [20].

### Agility

**Fig 2:**
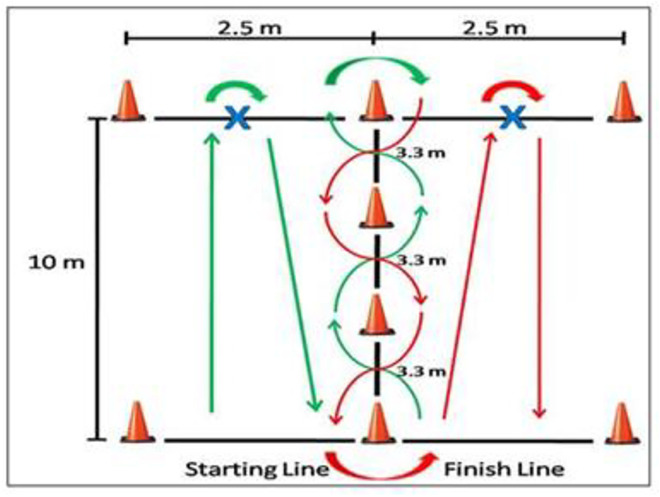
Illinois agility test [22].

The *“Illinois Agility Test”* was used to determine the agility performance of male handball players. As shown in Fig 2, a course was created with four cones arranged in a straight line for slalom, with a length of 10 m and a width of 5 m, with intervals of 3.3 m in between. Male handball players started the test by lying face down 30 cm behind the starting point of the course, with their hands in contact with the ground at shoulder level, and upon the verbal command of ‘start/go’, they stood up and began the test. The start and finish times were recorded in seconds using photoelectric cells. The test was conducted twice, and the best result was used for analysis [21–22].

### Data analysis

Data collected within the scope of the research were analyzed using the statistical program SPSS 29.0.2 (USA). Before the analysis, descriptive statistics for the variables of age, height, body weight, years of sports, agility, and vertical jump were generated. According to the results of the Shapiro-Wilk test (Age = .953, Height = .963, Body weight = .943, Years of sports = .950, Agility = .909, and Vertical jump = .925; p > 0.05) conducted for normality assumption, it was determined that the data showed a normal distribution. Therefore, the *“Pearson”* test was preferred for relationship analysis. Additionally, in order to clearly demonstrate the relationship between agility and vertical jump in the pr analysis; age, height, and body weight variables were controlled (α = 0.05). In this context, according to the absolute value of the Pearson correlation coefficient;.12 is considered a small relationship,.24 a medium relationship, and.41 a high relationship level [23].

## Results

The descriptive statistics of male handball players are shown in Table 1. In addition, the results of the correlation analysis are presented in Table 2 and Table 3.

**Table 1:** Descriptive statistical analysis table of male handball players.

Variables	N	Minimum	Maximum	Average	Standard deviation
Age (years)	15	17	35	24.1	5
Height (cm)		172	198	187.1	6.8
Body weight (kg)		63	130	88	16.8
Sports year (years)		4	22	11.3	4.7
Agility (sec.)		14.5	17.9	15.9	1.1
Vertical jump (cm)		62	93	73.5	9.3

N/n: Number of subjects

**Table 2:** Results of the relationship analysis of descriptive variables belonging to male handball players.

Variables		Height (cm)	Body weight (kg)	Sports year (years)	Agility (sec.)	Vertical jump (cm)
**Age (years)**	r	0.450	0.492	**.759** ^**^	0.175	**.657** ^**^
	p	0.093	0.063	0.001	0.534	0.008
**Height (cm)**	r		0.440	0.502	0.343	0.344
	p		0.101	0.057	0.210	0.209
**Body weight (kg)**	r			0.284	**.621** ^*^	0.208
	p			0.305	0.013	0.457
**Sports year (years)**	r				0.241	0.216
	p				0.387	0.439
**Agility (sec.)**	r					−0.298
	p					0.280

*p < 0.05; **p < 0.01

**Table 3:** Partial correlation between agility and vertical jump (controlling for age, height, and body weight).

Variables		Agility (sec.)	Vertical jump (cm)
**Age (years)**	pr	−0.557*	
	p	0.039	
**Height (cm)**	pr	−0.472	
	p	0.088	
**Body weight (kg))**	pr	−0.558*	
	p	0.038	

*p < 0.05

Looking at the average values in Table 1, it has been found that male handball players have an age of 24.1 ± 5 years, a height of 187.1 ± 6.8 cm, a body weight of 88 ± 16.8 kg, a sports career of 11.3 ± 4.7 years, agility of 15.9 ± 1.1 seconds, and a vertical jump of 73.5 ± 9.3 cm.

In Table 2, a high level of positive significant relationships were found between age and years of sport in male handball players (r = .759; p < 0.01) and vertical jump (r = .657; p < 0.01), while a high level of positive significant relationship was found between body weight and agility (r = .621; p < 0.05). There were no statistically significant relationships found among other variables (p > 0.05) (**H**_**1**_).

The result of the partial correlation analysis in Table 3 shows that when age (pr = −.557; p = 0.039) and body weight (pr = −.558; p = 0.038) are controlled, there is a highly significant negative relationship between agility and vertical jump. However, when height is controlled, no significant relationship was found between agility and vertical jump (p > 0.05) (**H**_**2**_).

## Discussion

This research aims to examine the relationship between sports performance characteristics such as agility and vertical jumping while controlling for developmental variables (age, height, and body weight). This is because developmental characteristics such as age and anthropometric factors can influence the scores among sports performance parameters [9]. According to the research findings, initially, no statistically significant relationship was found between agility and vertical jumping (r = −.298; p > 0.05) (**H**_**1**_). However, in the partial correlation analysis; when age (pr = −.557; p = 0.039) and body weight (pr = −.558; p = 0.038) were controlled, a significant negative relationship was found between agility and vertical jumping (**H**_**2**_). Therefore, this research highlights that the effect of developmental characteristics on sports performance should be taken into account. In the literature, similarly to this research, Sellami et al. [24] examined the sports performance parameters of athletes according to their maturity levels. In other studies, it has been observed that the sports performance characteristics of handball players [25] and basketball players [26] were analyzed based on their biological maturity status. Recently, it has been noted that biological maturity status is considered in sports performance studies. Therefore, although taking developmental characteristics into account is not a new approach, controlling for these variables in this research has enabled a more accurate interpretation of the relationship between agility and vertical jump performance in male handball players.

Looking at the final test results after high-intensity training applied to young male handball players, the experimental group (n = 17) jumped 40.6 ± 2.7 cm in the vertical jump test, while the control group (n = 15) jumped 31.8 ± 3.3 cm [27]. The pre-competition vertical jump height of young male handball players (N = 17; age 16.2 years) was measured at 34.6 ± 6.4 cm [28]. It was calculated that male handball players (N = 56; ages 12–14) jumped 30.42 ± 5.93 cm in the vertical jump test [8]. After the combined training applied to male handball players, the vertical jump values were 34.2 cm in the experimental group (n = 11, age 20.18 ± 1.32 years) and 34.7 cm in the control group (n = 11, age 22.09 ± 2.58 years) [29]. In the study by Hammami et al. [9], the jumping performance of male handball players in different age categories was determined as follows: U14 (n = 22) 27.8 ± 22.2 cm, U15 (n = 18) 27.8 ± 3.5 cm, U16 (n = 17) 30.1 ± 5.1 cm, U17 (n = 12) 32.7 ± 4.2 cm, and U18 (n = 10) 35.2 ± 2.7 cm. According to the research findings, the average vertical jump height has been measured at 73.5 ± 9.3 cm. Interpreting vertical jump performance based on absolute values without considering relative measurements like power produced in relation to body weight (e.g., Watt/kg) may pose a limitation in research [30]. This is because two athletes with the same jump height may have different body weights, leading to significant differences in neuromuscular power. Therefore, in this study, body weight, which is one of the developmental characteristics, has been evaluated as a control variable. The relationship between agility and vertical jump (pr = −.558; p = 0.038) emerged when body weight was considered as a control variable (**H**_**2**_). Additionally, while interpreting the findings of this research and the literature values regarding vertical jump performance, it has been observed that there is an increase in jump height with age. This increase cannot be reduced solely to chronological aging. As athletes age, they generally experience increases in muscle mass, neuromuscular coordination, and training experience. These characteristics contribute to force production and motor control and directly affect athletic performance. Furthermore, the evolution of technical skills and movement ability over time may play a significant role in the development of jumping ability [31–32].

As a result of different training applied to male handball players (N = 36, age 15−18 years), the Illinois test results were found to be 16.47 ± 1.34 seconds for the vertical core training group, 17.38 ± 1.27 seconds for the horizontal core training group, and 16.57 ± 0.82 seconds for the control group [33]. The modified Illinois test result was 12.46 ± 0.32 seconds for the experimental group and 13.06 ± 0.23 seconds for the control group [27]. In the study by Raya et al. [22], the agility test result for active male soldiers aged 18−40 was determined to be 18.18 ± 1.14 seconds. Among 117 young males (aged 14.95 ± 1.93 years) involved in different sports, the average values in the Illinois test were found to be 16.432 ± 0.591 seconds for football players, 16.655 ± 1.441 seconds for basketball players, 15.950 ± 0.615 seconds for volleyball players, and 16.632 ± 1.021 seconds for handball players [1]. According to the information conveyed by Mackenzie from Davis [21], the Illinois test’s national norm values for ages 16−19 are as follows: below 15.2 is excellent, 15.2–16.1 is above average, 16.2–18.1 is average, 18.2–18.3 is below average, and above 18.3 is poor. Based on this information, it is observed that the handball players participating in the research exhibit above-average agility performance (15.9 ± 1.1 sec). Therefore, it can be concluded that the handball players who participated in the study were well-trained during the competition season. Additionally, based on the studies examined, it was noted that agility performance is better in late childhood and young athletes. Hence, it is believed that agility performance will decline as age increases. Because, as age increases; there can be physiological changes in characteristics such as muscular atrophy, delay in neuromuscular responses, prolongation of reaction time, and flexibility [34]. Additionally, the decline in motor unit activation capacity with age and the decrease in the proportion of fast-twitch muscle fibers are factors that negatively affect agility performance [35].

Sellami et al. [24] stated in their study with football players (N = 647; ages 11–18 years) that agility and sprint tests have a significant correlation with muscle strength (vertical jump). In the study conducted by Hammami et al. [8], it is stated that anaerobic power characteristics such as jumping and sprinting are related to planned agility tests. The negative correlation between the planned agility test applied to male students (age 20.73 ± 1.26 years) and vertical jump indicates that vertical jump has a positive effect on agility [36]. Katsumata and Aoki [37] determined in their study that as the vertical jump height of elite male handball players (N = 51) increases, zig-zag agility performance also improves. In the study by Hermassi et al. [38] the vertical jump performance of male handball players (n = 70; age 15.8 ± 0.3 years) was determined to be 35.07 ± 7.44 cm, while their Illinois agility test performance was 8.28 ± 1.03 seconds. It was also stated in the same study that the agility test developed specifically for handball was statistically related to the vertical jump performance. In the study conducted by Hachana et al. [39], university students (N = 105, age 20.82 ± 1.31 years) involved in football, rugby, and handball had an Illinois agility test result of 16.30 ± 0.77 seconds and a vertical jump test result of 40.37 ± 5.20 cm. Similar to the research findings of this study, a negative correlation was found between the agility test results and the vertical jump test. In the study by Hammami et al. [9], the results of the jump test established negative significant relationships with the results of the modified Illinois agility test. When looking at the research findings, although it resembles studies in the literature, the relationship between agility and vertical jump emerged when age and body weight were controlled (**H**_**1**_, **H**_**2**_). Therefore, it is understood that developmental characteristics affect sports performance in the context of this research.

## Conclusion and suggestions

As a result, while the agility performance of male handball players is above average, the jump height is also at a high level. It has been understood from the studies examined that agility performance yielded better results in late childhood and adolescence athletes. In contrast, performance improvement in jump height occurs with increasing age. Additionally, the relationship between agility and vertical jump only emerged when developmental characteristics (age and height) were controlled. It is recommended that studies be conducted by controlling biological maturity level or the effects of developmental characteristics on sports performance. Future research based on this proposal can provide important insights into how maturation and developmental characteristics affect sport-related physical abilities over time. It is also recommended that trainers and coaches add training practices aimed at improving agility and vertical jump performance to exercise prescriptions and test these features at regular intervals throughout the competition season.

Additionally, this research has made a valuable/original contribution to the literature by examining the selected tests and the relationship between them, particularly in the context of developmental characteristics in handball sports. Therefore, this research could be an important reference source for coaches, trainers, and sport scientists aimed at determining athletic performance in handball and optimizing talent selection processes.

### Limitation of the research

In this research, evaluating the vertical jump height based on absolute values instead of normalized relative parameters (e.g., Newton/kg or Joule/kg) according to body weight is a limitation.
